# A Rare Case of Benign-Appearing Myoepithelial Carcinoma of the Hard Palate in an Adolescent

**DOI:** 10.3390/jcm15197708

**Published:** 2026-10-06

**Authors:** Eun Young Lee, Baljinnyam Altangerel, Kang Hee Yu

**Affiliations:** 1 Department of Oral and Maxillofacial Surgery, College of Medicine and Medial Research Institute, Chungbuk National University, Cheongju 28644, Republic of Korea; ley926@chungbuk.ac.kr (E.Y.L.); baljinnyam855@gmail.com (B.A.); 2 Department of Oral and Maxillofacial Surgery, Chungbuk National University Hospital, Cheongju 28644, Republic of Korea

**Keywords:** myoepithelial carcinoma, salivary gland neoplasm, palate, adolescent, case report

## Abstract

Myoepithelial carcinoma is a rare malignant salivary gland tumor that is particularly uncommon in pediatric patients. A 14-year-old girl presented with a painless, slowly enlarging hard palate mass that had been present for 2 years. Clinically, the lesion closely resembled a benign tumor due to its young presentation age, well-encapsulated state, absence of mucosal ulceration, and lack of lymphadenopathy, leading to an initial diagnosis of pleomorphic adenoma. Complete surgical excision with superficial mucosa preservation was initially performed. However, histopathological examination revealed myoepithelial carcinoma with infiltrative growth despite its well-circumscribed appearance. Consequently, a second-stage wide local excision was performed to achieve adequate safety margins. The patient recovered uneventfully without functional impairment, with preservation of oral mucosal mobility. No evidence of recurrence was observed during 18 months of follow-up. This case demonstrates that myoepithelial carcinoma may present as a clinically benign, well-circumscribed palatal mass even in pediatric patients, emphasizing that benign clinical features and a negative intraoperative frozen-section result do not exclude malignancy and highlighting the importance of careful permanent histopathological examination and long-term postoperative surveillance.

## 1. Introduction

Myoepithelial carcinoma is an uncommon malignant salivary gland neoplasm, accounting for less than 1% of all salivary gland tumors [1,2]. It most commonly arises in the parotid gland, followed by the submandibular gland, whereas involvement of the minor salivary glands is uncommon [2,3,4,5]. Myoepithelial carcinoma predominantly affects middle-aged and older adults, with a mean age at diagnosis of approximately 55 years [2,4]. Salivary gland tumors are uncommon in the pediatric population, and myoepithelial carcinoma is particularly rare in children and adolescents, with only a limited number of cases reported in the literature [6,7].

The precise etiology of myoepithelial carcinoma remains incompletely understood. It may arise de novo or in association with a pre-existing pleomorphic adenoma [1,4,8,9]. Histopathologically, myoepithelial carcinoma exhibits considerable morphological heterogeneity, with tumor cells displaying epithelioid, plasmacytoid, spindle, or clear-cell features and arranged in solid, nested, cord-like, or multinodular patterns [1,4,8,10]. Infiltrative growth into the surrounding tissues is a key feature supporting malignancy [1,4,5,8]. Immunohistochemically, tumor cells typically express epithelial markers together with myoepithelial markers, including S-100 protein, SOX10, p63, calponin, and smooth muscle actin [1,8]. Because of its broad morphological spectrum, the differential diagnosis includes pleomorphic adenoma, benign myoepithelioma, epithelial–myoepithelial carcinoma, adenoid cystic carcinoma, and other salivary gland neoplasms showing myoepithelial differentiation [1,8].

The clinical diagnosis of myoepithelial carcinoma is often challenging because its presentation may closely resemble that of benign salivary gland tumors. Slow growth, the absence of pain or ulceration, and a well-circumscribed appearance can lead to an initial diagnosis of pleomorphic adenoma, despite the presence of underlying malignant pathology. This diagnostic challenge is particularly pronounced in younger patients, in whom malignant salivary gland tumors are rarely suspected.

Myoepithelial carcinoma arising in the palate is rare [11]. Herein, we report a case involving a 14-year-old girl. The lesion exhibited clinical features suggestive of a benign salivary gland tumor, including a well-circumscribed appearance without mucosal ulceration or cervical lymphadenopathy, resulting in an initial diagnosis of pleomorphic adenoma. Histopathological examination after surgical excision, however, revealed infiltrative myoepithelial carcinoma, necessitating additional wide excision. This case highlights the diagnostic pitfalls of clinically benign-appearing palatal masses in pediatric patients and emphasizes the importance of maintaining suspicion for malignancy despite reassuring clinical findings [12].

## 2. Case

A 14-year-old girl presented to our hospital with a chief complaint of a painless swelling on the right side of the hard palate. She had first noticed the palatal mass approximately 2 years earlier, during which time it had gradually increased in size without associated pain, prompting her to seek medical evaluation.

The patient had no significant medical or family history and was a non-smoker. The review of systems was unremarkable, with no history of general weakness, fatigue, headache, dizziness, fever, chills, chest pain, dyspnea, abdominal pain, or urinary or bowel symptoms. The patient was alert, and the head and neck examination was otherwise unremarkable. Laboratory investigations revealed no significant abnormalities.

**Figure 1:**
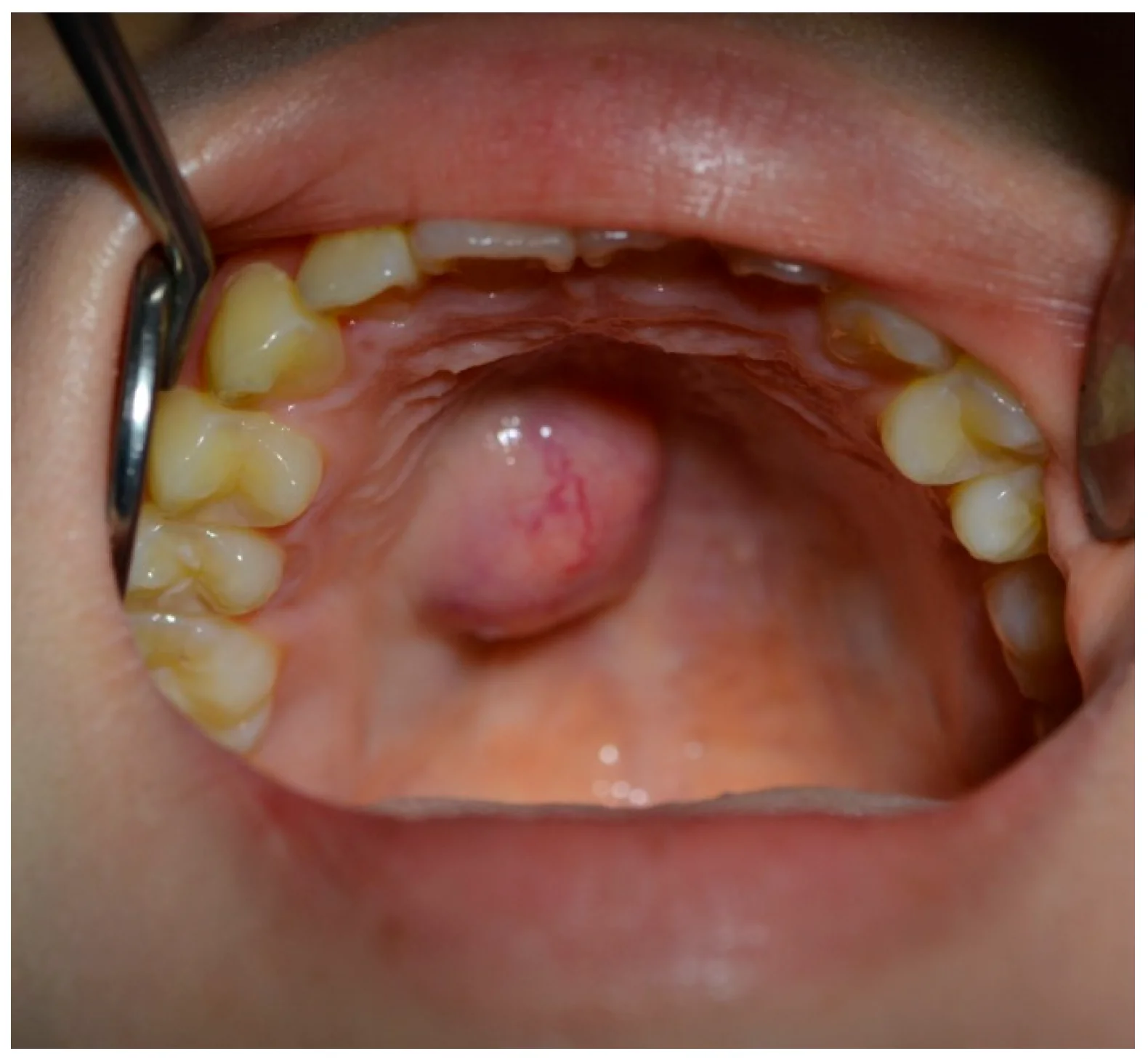
Clinical photograph showing a soft, elevated mass on the right anterior hard palate measuring 12 × 14 mm clinically.

Intraoral examination revealed a sessile, soft, well-defined submucosal mass measuring approximately 15 mm in diameter on the right hard palate. The lesion was associated with underlying bone resorption but showed no clinical evidence of invasion. The overlying mucosa was intact, with no ulceration or erosion, and the mass was fixed and non-tender (Figure 1).

**Figure 2:**
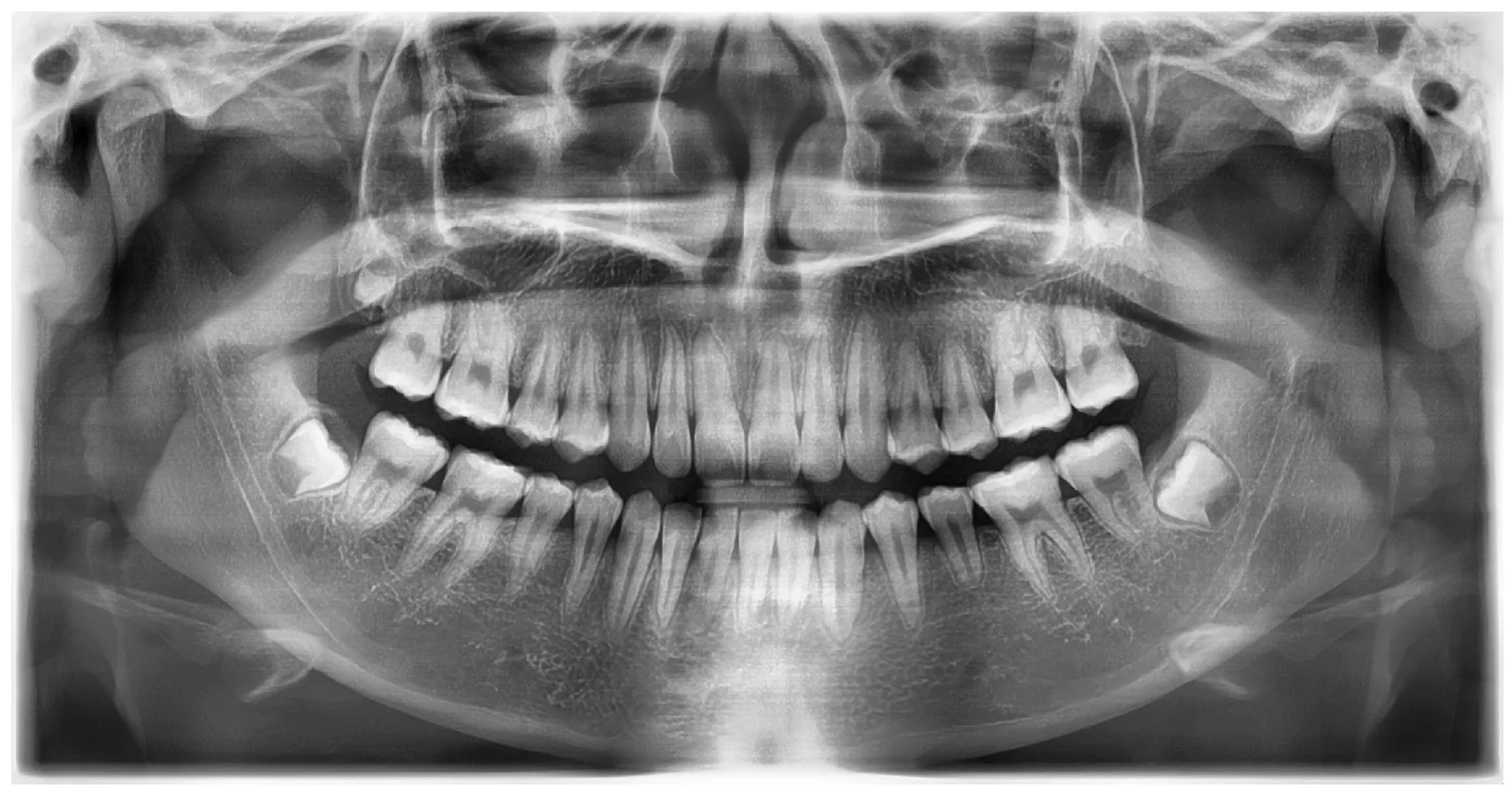
Initial panoramic radiograph demonstrating no remarkable osseous abnormalities.

**Figure 3:**
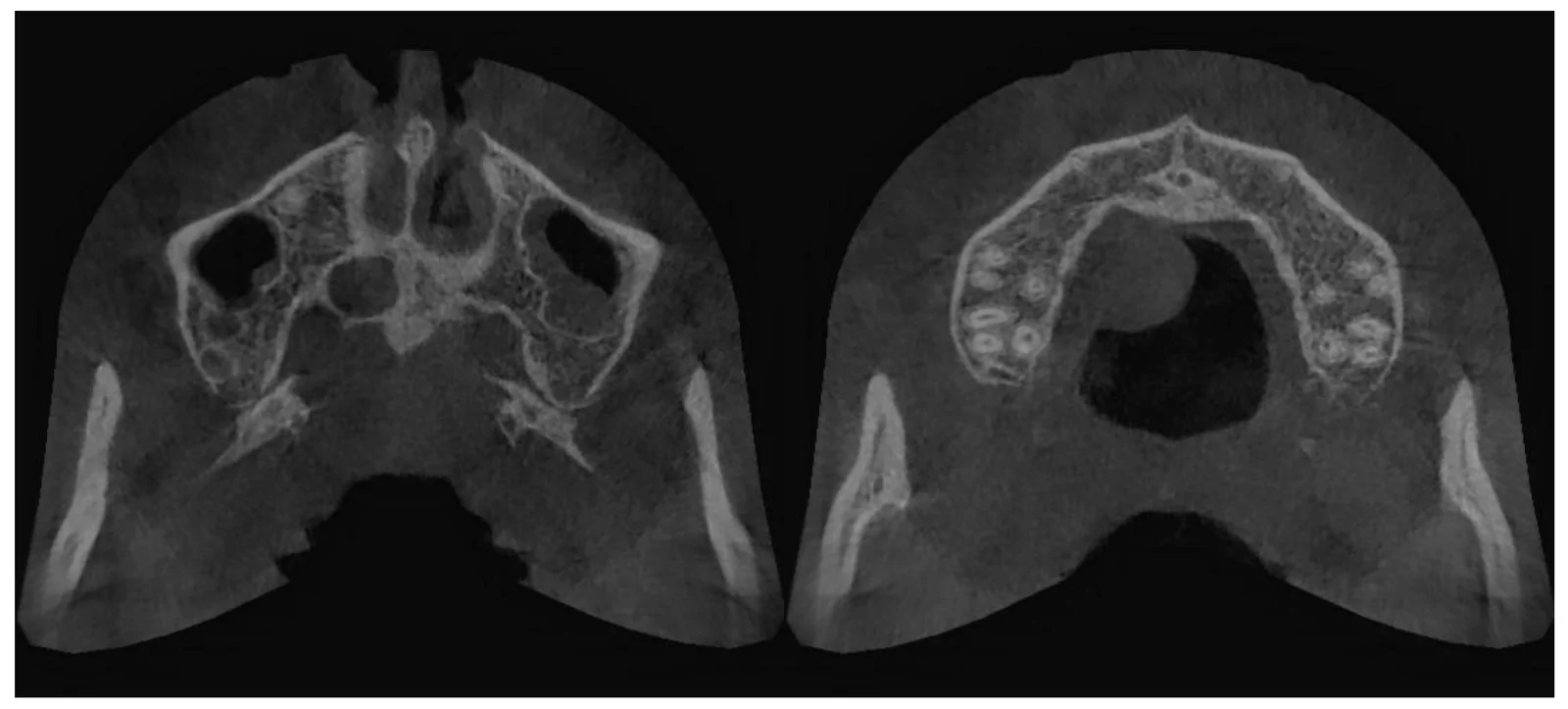
Axial CBCT image showing focal pressure resorption of the adjacent palatal bone without cortical destruction.

**Figure 4:**
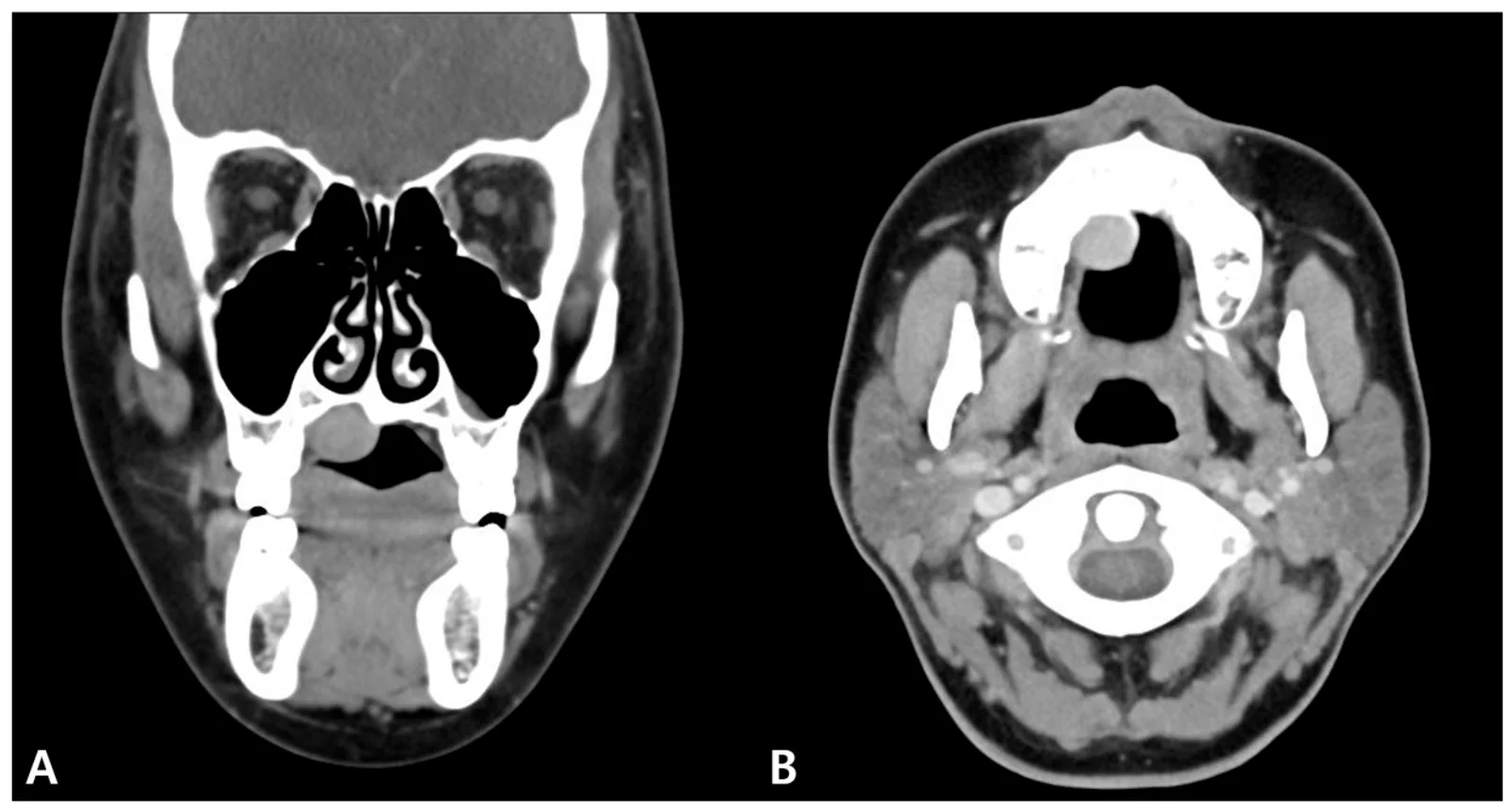
Contrast-enhanced CT images of the palatal lesion. (**A**) Coronal and (**B**) axial images demonstrate a well-circumscribed, enhancing soft-tissue mass in the right anterior hard palate, without evidence of infiltrative growth into the adjacent maxillary bone or surrounding soft tissues.

On the initial visit, diagnostic imaging, including panoramic radiography, cone-beam computed tomography (CBCT), and contrast-enhanced neck computed tomography (CT), was performed. Panoramic radiography did not demonstrate any remarkable findings (Figure 2). CBCT demonstrated focal pressure resorption of the adjacent palatal bone without cortical destruction or radiographic evidence of aggressive osseous invasion (Figure 3). Contrast-enhanced CT demonstrated a well-circumscribed, homogeneously enhancing soft tissue mass involving the right anterior hard palate without radiographic evidence of infiltration into the adjacent maxillary bone or surrounding soft tissues (Figure 4). No cervical lymphadenopathy was identified on contrast-enhanced neck CT. Overall, the radiographic findings favored a benign salivary gland tumor rather than an aggressive malignant neoplasm.

**Figure 5:**
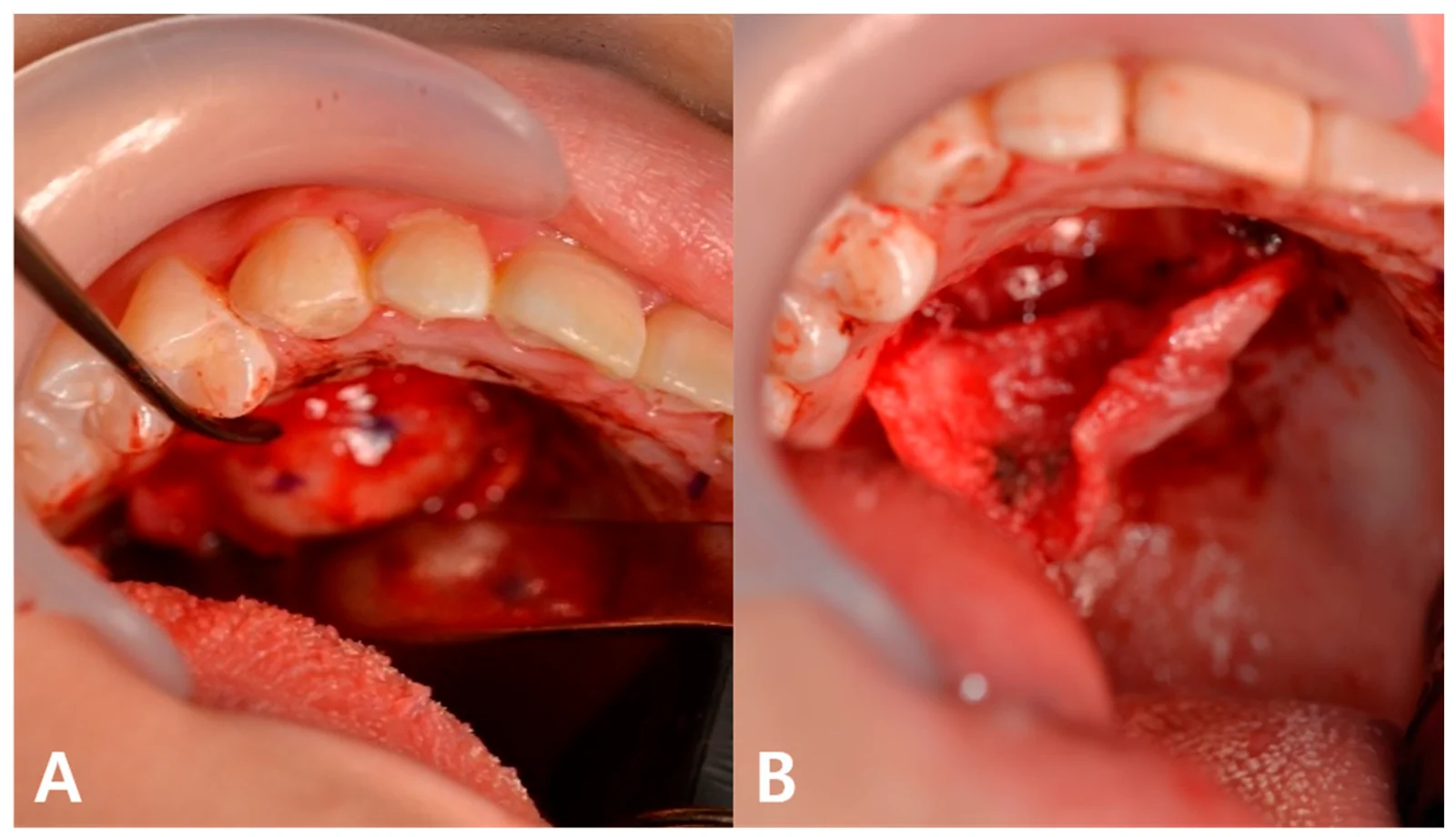
Intraoperative findings during the initial surgical excision. (**A**) The palatal mass exposed after dissection of the overlying mucosa. (**B**) Surgical site after complete excision of the mass with preservation of the overlying palatal mucosa.

**Figure 6:**
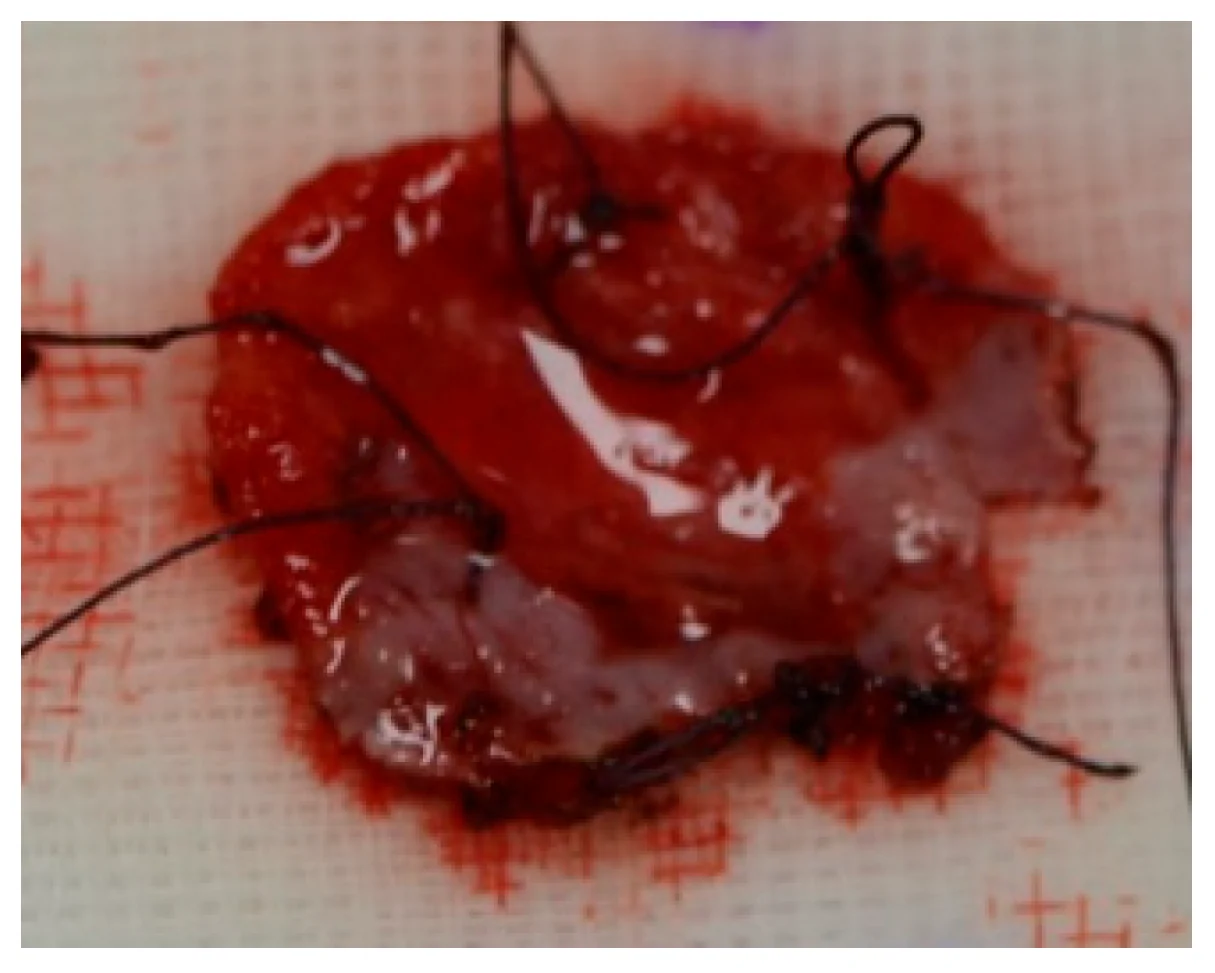
Gross appearance of the completely excised palatal mass.

Based on the clinical and radiographic findings, the lesion was initially diagnosed as a benign salivary gland neoplasm, most likely a pleomorphic adenoma. Given its small size, well-circumscribed appearance, intact overlying mucosa, and absence of radiographic features suggestive of malignancy, a separate preoperative incisional biopsy was not performed, and complete surgical excision was planned as both a diagnostic and therapeutic procedure. Under general anesthesia, a partial-thickness mucosal incision was made over the palatal lesion, followed by careful dissection while preserving the overlying palatal mucosa. Intraoperatively, the lesion appeared well encapsulated, and the underlying palatal bone showed compressive resorption without gross evidence of osseous invasion. The base of the lesion was firmly adherent to the adjacent palatal soft tissue. The lesion was completely excised (Figure 5), and intraoperative frozen-section examination showed no evidence of malignancy. The excised specimen was subsequently submitted for definitive histopathological examination (Figure 6). The surgical site was closed with 4-0 Vicryl^®^ sutures. Definitive histopathological examination subsequently revealed myoepithelial carcinoma with an infiltrative growth pattern.

**Figure 7:**
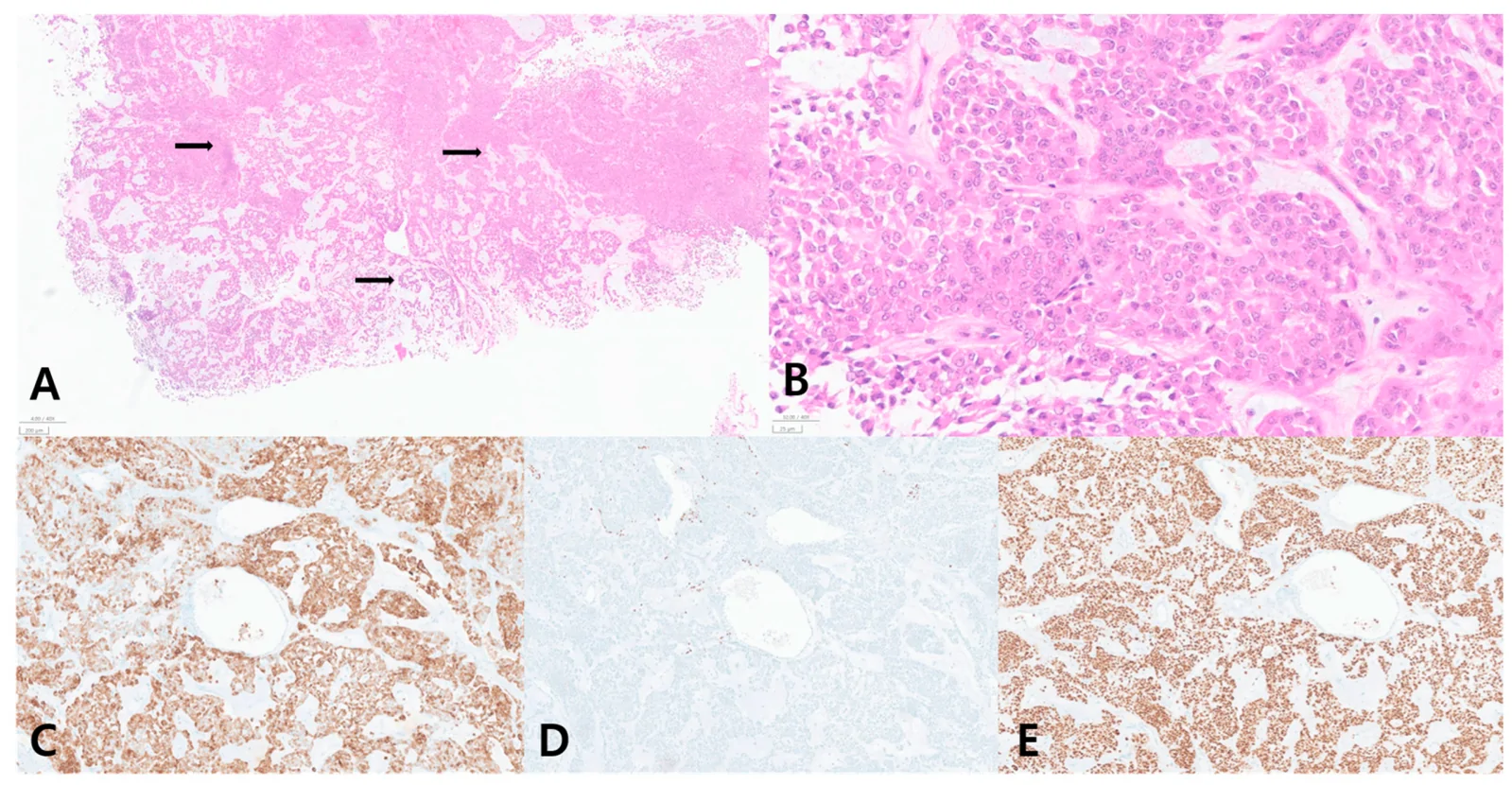
Histopathological and immunohistochemical findings of myoepithelial carcinoma. (**A**) Low-power H&E image showing an infiltrative multinodular growth pattern within the surrounding stroma. (**B**) High-power H&E image demonstrating plasmacytoid and epithelioid tumor cells arranged in sheets and nests. (**C**) CK7 immunohistochemistry showing heterogeneous expression in the predominant tumor-cell population as well as in focal ductular/luminal cells. (**D**) p63 immunohistochemistry showing focal, scattered nuclear positivity in tumor cells. (**E**) SOX10 immunohistochemistry showing diffuse nuclear positivity in the predominant tumor-cell population.

Definitive histopathological examination revealed an infiltrative multinodular proliferation of tumor cells beneath the intact surface epithelium (Figure 7A). The tumor was predominantly composed of plasmacytoid and epithelioid cells arranged in sheets and nests within a myxoid to mildly hyalinized stroma, with focal ductular formation (Figure 7B). The tumor cells exhibited mild-to-moderate nuclear pleomorphism and increased mitotic activity, with a mitotic count of 10 per 10 high-power fields, including atypical mitotic figures. No tumor necrosis, lymphovascular invasion, or perineural invasion was identified.

Immunohistochemically, SOX10 showed diffuse nuclear positivity in the predominant tumor-cell population, whereas p63 showed focal, scattered nuclear positivity. S-100 showed broad positivity in the predominant tumor-cell population. CK7 expression was present not only in focal ductular/luminal cells but also in a substantial proportion of the predominant tumor-cell population, with heterogeneous staining intensity. Focal p53 positivity was observed, and the Ki-67 proliferation index was approximately 10%. Representative immunohistochemical findings for CK7, p63, and SOX10 are shown in Figure 7C–E. Based on these morphological and immunohistochemical findings, a diagnosis of myoepithelial carcinoma, plasmacytoid type, was established.

Although the initial resection margins were histologically negative, the minimum microscopic clearance was only 2 mm. Because the initial excision had been performed under the clinical impression of a benign salivary gland neoplasm and the permanent specimen unexpectedly demonstrated myoepithelial carcinoma with an infiltrative growth pattern, the 2 mm clearance was considered insufficient for definitive oncologic management.

**Figure 8:**
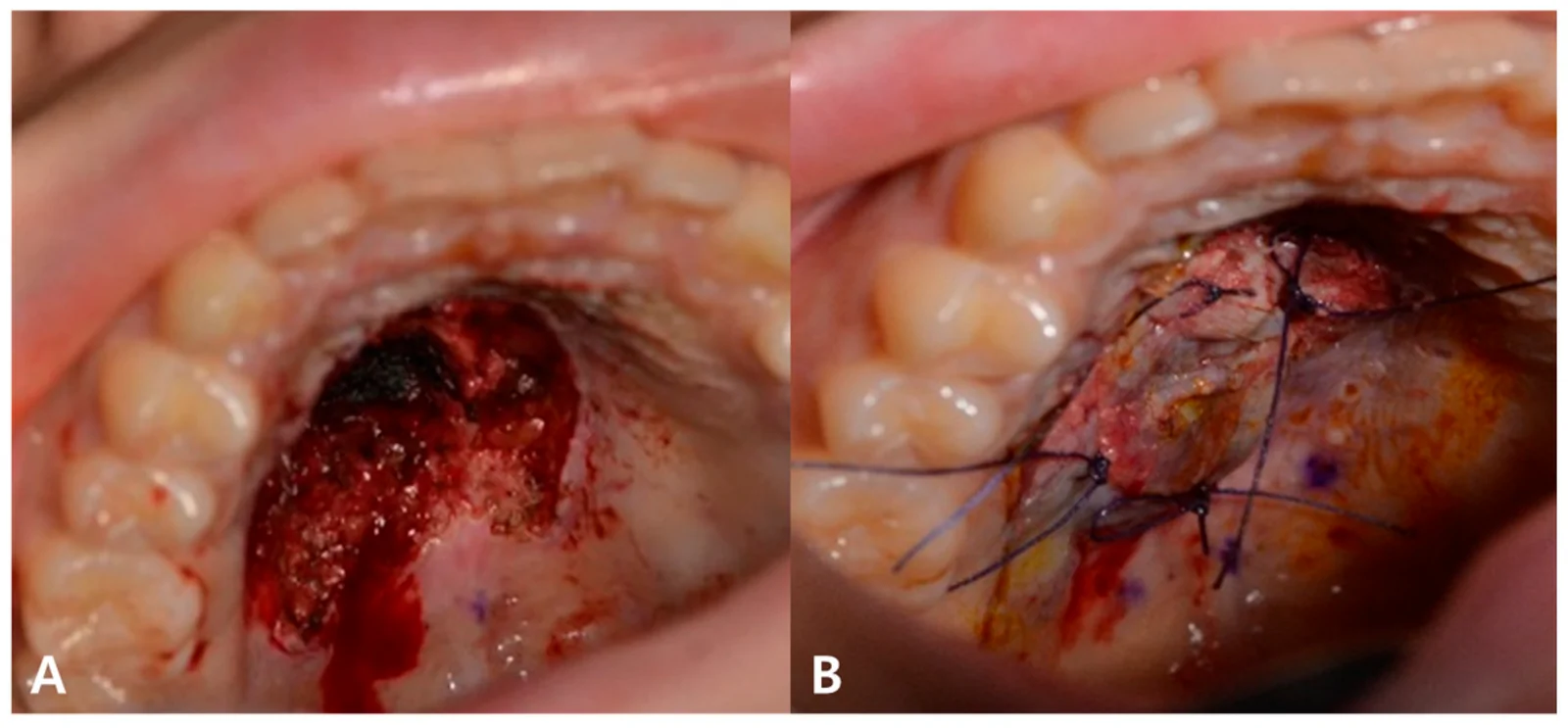
Second-stage surgery. (**A**) Wide local excision of the previous surgical site with an approximately 5 mm gross surgical margin. (**B**) The surgical defect was covered with fibrin glue and Vaseline gauze and allowed to heal by secondary intention without additional reconstructive procedures.

Therefore, a second-stage wide local excision of the previous surgical site was performed with an approximately 5 mm gross surgical margin on both the palatal and posterior soft-tissue aspects (Figure 8). Additional specimens were obtained from the posterior, right posterior, left posterior, and left posterior bony aspects. No flap, graft, or other reconstructive procedure was performed. The resulting defect was covered with fibrin glue and Vaseline gauze and allowed to heal by secondary intention. As there was no gross evidence of osseous invasion, extensive resection of the hard-palate bone was not performed; only a small portion of suspicious palatal bone was removed and submitted for histopathological examination. Histopathological examination of the re-excision specimens revealed no residual tumor, and all examined resection margins were negative. Because no residual tumor was identified, a microscopic tumor-to-margin distance could not be determined.

Following the diagnosis of myoepithelial carcinoma, postoperative CBCT and PET/CT were performed for staging evaluation. CBCT showed no evidence of invasive osseous involvement, and PET/CT demonstrated no evidence of regional lymph node or distant metastasis. Because depth of invasion was not documented in the original histopathological report, a definitive pathological T category according to the AJCC 8th edition oral cavity staging system could not be assigned retrospectively; clinically, there was no evidence of regional or distant metastatic disease (cN0 cM0).

**Figure 9:**
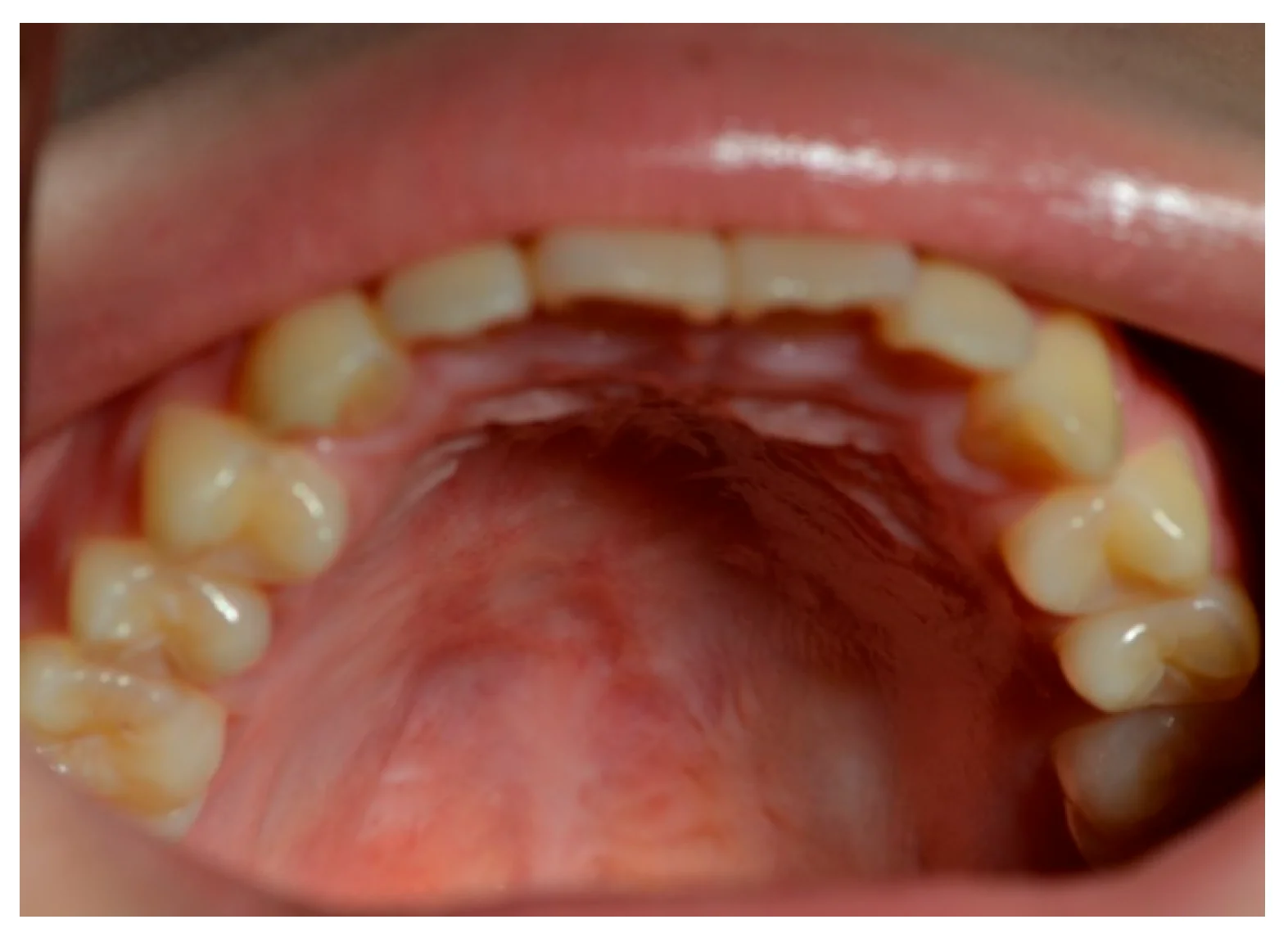
Clinical photograph obtained 18 months after the second-stage surgery demonstrating complete healing of the palatal mucosa with preservation of mucosal mobility and no evidence of local recurrence.

Following consultation with the Department of Radiation Oncology, adjuvant radiotherapy was not recommended. Although the tumor demonstrated an infiltrative growth pattern and increased mitotic activity, the absence of residual tumor after re-excision, clear surgical margins, and the absence of lymphovascular invasion, perineural invasion, tumor necrosis, and osseous invasion were considered together with the potential long-term morbidity of radiotherapy in this 14-year-old patient. Close surveillance was therefore selected. The patient was scheduled for regular postoperative surveillance to monitor for local recurrence or distant metastasis, with follow-up visits every 3 months during the first postoperative year and every 6 months thereafter. At the 18-month follow-up, positron emission tomography/computed tomography (PET/CT) demonstrated no evidence of local recurrence, regional lymph node metastasis, or distant metastatic disease. The patient remained free of functional impairment, with satisfactory wound healing and preserved palatal mucosal function throughout the follow-up period (Figure 9).

## 3. Discussion

Myoepithelial carcinoma occurs predominantly in the parotid gland and primarily affects middle-aged and older adults, with a mean age at presentation of approximately 55 years and no clear sex predilection [2,4]. Pediatric and adolescent myoepithelial carcinoma is exceptionally rare, with only a limited number of cases reported in the literature [7,13]. Morinière et al. reported myoepithelial carcinoma of the parotid gland in an 8-year-old girl [13], and another pediatric case involving the parotid gland in an 8-year-old girl was recently reported by Prathibha et al. [7]. In contrast to these pediatric cases involving the major salivary glands, the present tumor arose from a minor salivary gland of the hard palate in a 14-year-old girl. Thus, the combination of the patient’s young age and the unusual palatal location represents a particularly uncommon presentation of myoepithelial carcinoma and contributed substantially to the diagnostic difficulty in the present case.

Previously reported cases of palatal myoepithelial carcinoma are summarized in Table 1. The present case differs from most previous reports in several important respects. Most previously reported palatal cases occurred in middle-aged or older adults, whereas our patient was only 14 years old [14,15,16,17,18]. In addition, several reported cases presented with mucosal ulceration, relatively large tumors, destructive bone involvement, or extension into adjacent structures [14,15,16,17,18]. In contrast, the present lesion was small (1.6 × 1.4 cm), slowly enlarged over approximately 2 years, remained covered by intact palatal mucosa, and showed only pressure resorption of the underlying palatal bone without destructive osseous invasion. Despite these clinically and radiographically benign-appearing features, definitive histopathological examination demonstrated an infiltrative multinodular growth pattern with increased mitotic activity. Thus, the exceptionally young age of the patient and the marked discrepancy between the benign clinical presentation and malignant histopathological findings distinguish the present case from most previously reported palatal cases.

Among the previously reported cases, that of Koike et al. provides a particularly relevant diagnostic comparison. Their 83-year-old patient presented with a 17 × 15 mm palatal lesion with mucosal ulceration and no radiographic evidence of bone invasion; an initial biopsy was diagnosed as myoepithelioma, whereas examination of the resected specimen revealed myoepithelial carcinoma [17]. Similarly, intraoperative frozen-section examination in our patient showed no evidence of malignancy, whereas permanent histopathological examination demonstrated infiltrative myoepithelial carcinoma. This discrepancy underscores the limitations of frozen-section examination in lesions with deceptively benign clinical features and highlights that a negative frozen-section result does not exclude malignancy; definitive diagnosis requires careful evaluation of adequately sampled permanent sections.

However, our patient was considerably younger and showed neither mucosal ulceration nor destructive bone invasion. At the other end of the clinical spectrum, Fang et al. reported a 65-year-old patient with a large hard-palate myoepithelial carcinoma measuring approximately 7 cm on CT, with mucosal ulceration and necrosis, extensive bone involvement, and subsequent pulmonary metastases [18]. In contrast, our patient showed no clinically aggressive features and remained free of recurrence or metastasis at 18 months following second-stage wide local excision without adjuvant radiotherapy. These comparisons illustrate the broad clinical spectrum of palatal myoepithelial carcinoma and emphasize that malignant behavior cannot be excluded solely on the basis of an indolent clinical or radiographic appearance. A limitation of this report is the relatively short follow-up period of 18 months, which is insufficient to fully assess long-term oncological outcomes. Therefore, continued long-term surveillance for local recurrence and distant metastasis is warranted.

Myoepithelial carcinoma has a substantial tendency toward local recurrence, and recurrent disease has been reported in several clinicopathological series [2,4,5,10]. This tendency may be related to its infiltrative growth pattern and the presence of an incomplete or deceptively well-defined capsule-like boundary, which can result in inadequate initial excision.

The differential diagnosis of myoepithelial carcinoma includes several benign and malignant salivary gland neoplasms, particularly pleomorphic adenoma, benign myoepithelioma, epithelial–myoepithelial carcinoma, adenoid cystic carcinoma, and mucoepidermoid carcinoma [19]. In the present case, pleomorphic adenoma was the principal initial consideration because of the slow growth and benign clinical appearance of the lesion. However, definitive diagnosis required careful histopathological and immunohistochemical evaluation.

Histologically, myoepithelial carcinoma may resemble pleomorphic adenoma because both tumors can contain myoepithelial cells and myxoid or hyalinized stromal components. However, infiltrative growth is a key feature distinguishing myoepithelial carcinoma from benign myoepithelial neoplasms, while multinodular architecture, cytologic atypia, and increased mitotic activity may provide additional diagnostic support [1,4,10]. A zonal growth pattern, characterized by greater cellularity at the periphery of tumor nodules and relative hypocellularity in their central portions, has also been described in myoepithelial carcinoma [1]. In the present case, the diagnosis was further supported by immunohistochemical positivity for S-100 protein, SOX10, p63, and CK7, in conjunction with the characteristic morphology and infiltrative growth pattern.

Epithelial–myoepithelial carcinoma is an important histological differential diagnosis, particularly because focal ductular formation may also be observed in myoepithelial carcinoma. Epithelial–myoepithelial carcinoma characteristically demonstrates a biphasic tubular architecture composed of an inner layer of eosinophilic ductal epithelial cells surrounded by an outer layer of clear myoepithelial cells [8,20]. In the present case, although focal ductular formation was identified, the characteristic biphasic tubular architecture of epithelial–myoepithelial carcinoma was absent. Instead, the tumor predominantly consisted of plasmacytoid and epithelioid myoepithelial cells arranged in sheets and nests with an infiltrative multinodular growth pattern.

Immunohistochemically, SOX10 and p63 positivity supported myoepithelial differentiation, whereas CK7 positivity was interpreted in conjunction with the overall morphological findings [1,8]. Taken together with the predominant plasmacytoid and epithelioid morphology and infiltrative multinodular growth pattern, these findings favored myoepithelial carcinoma over epithelial–myoepithelial carcinoma. Molecular alterations involving HRAS have been frequently reported in epithelial–myoepithelial carcinoma and may provide additional diagnostic support in selected challenging cases [20]. Molecular analysis was not performed in the present case because the diagnosis could be established based on the morphological and immunohistochemical findings.

Mucoepidermoid carcinoma was also considered in the differential diagnosis because focal cystic and duct-like structures were present. However, the absence of a characteristic combination of mucous, intermediate, and epidermoid cells argued against mucoepidermoid carcinoma [8]. In contrast, the plasmacytoid and spindle-shaped tumor cells, infiltrative multinodular growth, and immunopositivity for myoepithelial markers supported the diagnosis of myoepithelial carcinoma.

Comparison with pleomorphic adenoma and mucoepidermoid carcinoma is clinically relevant because these tumors may exhibit overlapping clinical or histological features (Table 2). Distinguishing them is essential, as their surgical management and expected clinical behavior differ substantially. In particular, failure to recognize myoepithelial carcinoma may lead to conservative excision with insufficient margins and consequently increase the risk of local recurrence.

Several reports have described malignant salivary gland tumors that were initially considered to be pleomorphic adenomas, resulting in delayed definitive treatment or inadequate initial surgery [23]. These observations emphasize that a slowly growing, well-circumscribed salivary gland mass should not automatically be regarded as benign, particularly when it occurs at an unusual site or demonstrates adhesion to adjacent tissue.

In many cases, the definitive diagnosis of myoepithelial carcinoma is established only after permanent histopathological examination of the excised specimen. Intraoperative frozen-section examination may assist in distinguishing benign from malignant disease and in evaluating surgical margins; however, its diagnostic accuracy can be limited by sampling error and the heterogeneous morphology of salivary gland tumors. A previous report described successful intraoperative diagnosis using frozen-section examination, subsequently confirmed on permanent paraffin sections [9]. In the present case, however, the frozen-section specimen showed no evidence of malignancy, whereas examination of the permanent specimen revealed infiltrative myoepithelial carcinoma. This discrepancy illustrates that a negative frozen-section result does not completely exclude malignancy when the clinical or intraoperative findings remain suspicious.

## 4. Conclusions

This case highlights that myoepithelial carcinoma of the minor salivary glands can closely mimic a benign salivary gland neoplasm, such as pleomorphic adenoma, particularly in young patients with a slowly growing, well-circumscribed lesion. Despite its benign clinical and radiographic appearance, the tumor demonstrated an infiltrative growth pattern on definitive histopathological examination. Careful histopathological evaluation with adequate tissue sampling and immunohistochemical analysis is therefore essential for accurate diagnosis. Awareness of this diagnostic challenge is important to ensure appropriate surgical management and long-term surveillance for local recurrence.

## Figures and Tables

**Table 1:** Comparison of the present case with selected previously reported cases of palatal myoepithelial carcinoma. * Malignant myoepithelioma is a historical term used in some earlier reports for myoepithelial carcinoma. Abbreviations: F, female; M, male; NR, not reported; HPF, high-power field.

Study	Age/ Sex	Size/ Duration	Clinical and Radiographic Features	Diagnosis/ Histopathology
Patrocinio et al., 2009 [14]	38/M	NR/ several years	Nasal obstruction, rhinorrhea, recurrent epistaxis; extension to nasal cavity and maxillary sinus	Malignant myoepithelioma *
Acikalin et al., 2009 [15]	79/F	3 × 4 cm/ 4 months	Palatal mass; initially diagnosed as poorly differentiated carcinoma on biopsy	Plasmacytoid and spindle cells with infiltrative growth
Richa et al., 2012 [16]	50/F	6 × 5 cm/ 2 years	Painless enlarging ulcerated mass; no radiographic bone erosion	Malignant plasmacytoid myoepithelioma *
Koike et al., 2015 [17]	83/F	17 × 15 mm/ ~3 months	Painless swelling with mucosal ulceration; no bone invasion; initial biopsy diagnosed as myoepithelioma	Myoepithelial carcinoma arising from myoepithelioma
Fang et al., 2019 [18]	65/M	7.0 × 6.6 × 7.5 cm/ 7 months	Large friable mass with ulceration/necrosis; extensive bone involvement and nasal extension	Clear-cell myoepithelial carcinoma
Present case	14/F	1.6 × 1.4 cm (pathological)/ ~2 years	Painless, slowly growing, intact mucosa; pressure resorption without destructive bone invasion; clinically suspected pleomorphic adenoma	Plasmacytoid myoepithelial carcinoma; infiltrative multinodular growth; 10 mitoses/10 HPFs

**Table 2:** Histopathological and immunohistochemical features in the differential diagnosis of myoepithelial carcinoma [8,19,21,22]. **Note:** Immunohistochemical profiles may vary among tumors and should be interpreted in conjunction with histomorphological features rather than used as isolated diagnostic criteria.

Feature	Pleomorphic Adenoma	Muco- Epidermoid Carcinoma	Epithelial– Myoepithelial Carcinoma	Myoepithelial Carcinoma
Histologic architecture	Mixed epithelial/myoepithelial elements with chondromyxoid stroma	Mixture of mucous, intermediate, and epidermoid cells; variable cystic architecture	Characteristic biphasic tubules with inner ductal and outer clear myoepithelial cells	Predominantly myoepithelial proliferation; solid, nested, cord-like, or multinodular growth
Cytomorphology	Variable myoepithelial morphology	Mucous, intermediate, epidermoid cells	Inner eosinophilic ductal cells and outer clear myoepithelial cells	Epithelioid, plasmacytoid, spindle, or clear cells
Growth pattern	Usually circumscribed, although pseudopodial extension may occur	Variable according to grade	Usually multinodular/infiltrative	Infiltrative growth supports malignancy
Immunophenotype	Epithelial + myoepithelial markers	Cytokeratins/p63; mucin-associated markers depending on cell type	CK7 in inner epithelial cells; p63/SOX10 in outer myoepithelial cells	Myoepithelial markers including SOX10, S100 and p63; keratin expression may occur
Key distinction	Well Chondromyxoid stroma without malignant infiltrative myoepithelial proliferation	Characteristic three-cell population	Distinct biphasic tubular architecture	Predominantly myoepithelial malignant proliferation without characteristic EMC biphasic architecture

## Data Availability

The data supporting the findings of this case report are not publicly available due to patient privacy and ethical restrictions.

## Abbreviations

The following abbreviations are used in this manuscript:

CBCT	Cone-Beam Computed Tomography
CT	Computed Tomography
H&E	Hematoxylin and Eosin
PET/CT	Positron Emission Tomography/Computed Tomography

## References

[1] Xu B., Katabi N. (2021). Myoepithelial Carcinoma. Surg. Pathol. Clin..

[2] Lavareze L., Scarini J.F., de Lima-Souza R.A., Kimura T.d.C., Gondak R.d.O., Egal E.S.A., Altemani A., Mariano F.V. (2023). Clinicopathological and survival profile of patients with salivary gland myoepithelial carcinoma: A systematic review. J. Oral Pathol. Med..

[3] Karatzanis A.D., Drivas E.I., Giannikaki E.S., Lachanas V.A., Hatziioannou J.K., Velegrakis G.A. (2005). Malignant myoepithelioma arising from recurrent pleomorphic adenoma of the soft palate. Auris Nasus Larynx.

[4] Savera A.T., Sloman A., Huvos A.G., Klimstra D.S. (2000). Myoepithelial carcinoma of the salivary glands: A clinicopathologic study of 25 patients. Am. J. Surg. Pathol..

[5] Kane S.V., Bagwan I.N. (2010). Myoepithelial Carcinoma of the Salivary Glands: A Clinicopathologic Study of 51 Cases in a Tertiary Cancer Center. Arch. Otolaryngol. Head Neck Surg..

[6] Bashar M.A., Shoab A.M. (2025). Salivary Gland Tumours in Children and Adolescents: A 60 Case Study. Int. J. Otolaryngol. Res..

[7] Prathibha R., Suresh T.N., Shilpa M.D., Sneha K., Sagayaraj A. (2025). Childhood Conundrum: A Rare Encounter with Myoepithelial Carcinoma of the Parotid Gland in an 8-Year-Old Girl. J. Appl. Health Sci. Med..

[8] Thompson L.D.R., Xu B. (2023). Top Ten Differentials to Mull Over for Head and Neck Myoepithelial Neoplasms. Head Neck Pathol..

[9] Cormier C., Agarwal S. (2022). Myoepithelial Carcinoma Ex-Pleomorphic Adenoma: A Rare Pathology Misdiagnosed as Pleomorphic Adenoma; With a Novel TERT Promoter Mutation and High PD-L1 Expression. Head Neck Pathol..

[10] Wang C., Zhang Z., Ge Y., Liu Z., Sun J., Gao Z., Li L. (2015). Myoepithelial Carcinoma of the Salivary Glands: A Clinicopathologic Study of 29 Patients. J. Oral Maxillofac. Surg..

[11] Lata J., Ahmad F., Chand V. (2014). Myoepithelial carcinoma of buccal mucosa: A rare tumor. Contemp. Clin. Dent..

[12] Chhieng D.C., Paulino A.F. (2002). Cytology of myoepithelial carcinoma of the salivary gland. Cancer.

[13] Morinière S., Robier A., Machet M.C., Beutter P., Lescanne E. (2003). Massive infra-clinic invasion of the facial nerve by a myoepithelial carcinoma of the parotid. Int. J. Pediatr. Otorhinolaryngol..

[14] Patrocinio L.G., Damasceno P.G., Patrocinio J.A. (2009). Malignant myoepithelioma of the hard palate: 9-year follow-up. Braz. J. Otorhinolaryngol..

[15] Acikalin M.F., Pasaoglu O., Cakli H., Gürbüz K., Canaz F. (2009). Malignant myoepithelioma of the palate: A case report with review of the clinicopathological characteristics. Yonsei Med. J..

[16] Richa, Ray J.G., Mohanty S.P., Vibha (2012). Malignant myoepithelioma of palate. Contemp. Clin. Dent..

[17] Koike T., Nariai Y., Hideshima K., Kondo S., Sekine J. (2015). A case of myoepithelial carcinoma in the palate derived from myoepithelioma. Jpn. J. Oral Maxillofac. Surg..

[18] Fang J., Kornfield A., Clavijo A., Vyas S., Ulbrandt A., Schroeder C., Byrd J.K., Kleven D. (2019). Clear Cell Myoepithelial Carcinoma Arising from the Hard Palate with Metastasis to the Lungs. Case Rep. Pathol..

[19] Compagno J., Wong R.T. (1977). Intranasal Mixed Tumors (Pleomorphic Adenomas): A Clinicopathologic Study of 40 Cases. Am. J. Clin. Pathol..

[20] El Hallani S., Udager A.M., Bell D., Fonseca I., Thompson L.D.R., Assaad A., Agaimy A., Luvison A.M., Miller C., Seethala R.R. (2018). Epithelial-Myoepithelial Carcinoma: Frequent Morphologic and Molecular Evidence of Preexisting Pleomorphic Adenoma, Common: HRAS: Mutations in: PLAG1-: Intact and: HMGA2-: Intact Cases, and Occasional: TP53, FBXW7, and: SMARCB1: Alterations in High-grade Cases. Am. J. Surg. Pathol..

[21] Seethala R.R. (2009). An update on grading of salivary gland carcinomas. Head Neck Pathol..

[22] Mejía-Hernández I.J., Cano Valdez A.M., De León-Trenado D., Luna-Ortíz K. (2013). Malignant myoepithelioma of the soft palate. Auris Nasus Larynx.

[23] Li Y., Wang Q. (2025). Myoepithelial carcinoma of submaxillary gland: A case report. Oncol. Lett..

